# From hearing loss to neurological complications: Is there an “ear glymphatic system”?

**DOI:** 10.1111/cns.14846

**Published:** 2024-08-21

**Authors:** Yanlin Bi, Yunchao Yang, Gaofeng Zhang, Hong Wang, Rui Dong

**Affiliations:** ^1^ Department of Anesthesiology Qingdao Municipal Hospital (Qingdao Hospital, University of Health and Rehabilitation Sciences) Qingdao China; ^2^ Department of Pediatrics, Qingdao Women and Children's Hospital Qingdao University Qingdao China; ^3^ Key Laboratory of Anesthesiology and Resuscitation (Huazhong University of Science and Technology) Ministry of Education Wuhan China

Presbycusis, also known as age‐related hearing loss, is a sensorineural hearing loss caused by degeneration of the auditory system with increasing age. Approximately 900 million older adults are predicted to suffer severe hearing impairment due to presbycusis by 2050. Presbycusis is strongly associated with depression, social isolation, frailty, cognitive decline, and even dementia. Undoubtedly, presbycusis is becoming a major public health burden, and further insights into its pathogenesis and novel avenues of effective therapeutic intervention are urgently required. A recent paper in *CNS Neuroscience & Therapeutics* by Xu et al. entitled “Evaluation of glymphatic system activity by diffusion tensor image analysis along the perivascular space in presbycusis” employs diffusion tensor imaging along the perivascular space (DTI‐ALPS) to assess glymphatic system activity in presbycusis patients.^1^ This study found a positive correlation between reduced glymphatic activity and cognitive decline, suggesting that glymphatic system dysfunction may contribute to the cognitive impairments seen in age‐related hearing loss. We were also intrigued by Shen et al.'s article in the *Journal of Clinical Anesthesia*,^2^ reveals a higher incidence of postoperative emergence agitation (POEA) in those with severe preoperative hearing impairment. The intersection of these studies presents a compelling hypothesis: glymphatic system dysfunction in hearing‐impaired elderly may contribute to the higher occurrence of POEA.

The glymphatic system in the brain is a waste disposal mechanism that manages the inflow and outflow of cerebrospinal fluid(CSF), facilitating the exchange of fluids and the clearance of metabolic waste, thus playing a crucial role in regulating fluid movement, waste removal, and potentially brain immunity.^3^ Diminished glymphatic function may be a hallmark of a vulnerable brain, and previous studies demonstrated that glymphatic deficiency aggravates β‐amyloid accumulation^4^ and impairs abnormal tau clearance.^5^ Hearing impairment could potentially trigger or exacerbate cognitive deficits by β‐amyloid and tau accumulation.^6^, ^7^ Combined with the study by Xu et al.,^1^ we presume that glymphatic system dysfunction is an important mechanism of cognitive dysfunction in patients with hearing impairment.

POEA is characterized by a state of confusion, agitation, and disorientation during the immediate recovery phase after anesthesia/operation. POEA and postoperative delirium (POD) have similar clinical features and risk factors. POD occurs due to the vulnerability of brain functioning to pathophysiological stressors, and associated with the level of β‐amyloid and tau in the CSF.^8^ Blunted glymphatic transport would cause the imbalances of immune homeostasis, making it more vulnerable to insults.^3^ Therefore, POEA could be influenced by impaired glymphatic function, leading to inadequate clearance of neurotoxic waste, which in turn leads to abnormal neurological agitation. Xu et al.'s study^1^ gave us reason to believe that glymphatic system dysfunction could be a contributing factor to the increased risk of POEA in elderly patients with hearing impairment. Further research is required to confirm this association and explore therapeutic strategies to enhance glymphatic function and auditory processing in the elderly undergoing surgery.

The inner ear's sensory hair cells are key players in presbycusis. These cells are responsible for converting sound vibrations into electrical signals, which are then transmitted to the brain. As individuals age, inner ear's sensory hair cells can undergo gradual wear and tear, lose their efficiency, or get damaged, leading to a decline in hearing ability. The inner ear's sensory hair cells are metabolically active and separated from the systemic circulation by the blood–labyrinth barrier, it remains unclear how inner ear's sensory hair cells effectively removing metabolic waste products. Additionally, the ear, similar to the brain and eye, has no traditional lymphatic vessels. The identification of ocular glymphatic clearance system suggests that the ear might also utilize CSF transport for the expulsion of metabolic waste.^9^ The study by Xu et al. employing DTI‐ALPS, revealed damage to the brain's glymphatic system in presbycusis patients,^1^ yet it remains unclear whether a similar glymphatic system exists in the ear. If further work confirms the presence of an “ear glymphatic system,” the implications would be wide‐ranging. The “ear glymphatic system” could explain the link between hearing loss and neurological disorders, offering a new perspective on how auditory health impacts overall neurological function. Mathiesen et al. showed that the cochlear aqueduct in mice exhibits lymphatic‐like characteristics, and large‐particle CSF tracers reached the inner ear by dispersive transport via the cochlear aqueduct in adult mice.^10^ Furthermore, they showed that viral constructs injected into the cisterna magna could successfully be delivered to the ear through the cochlear aqueduct and can restore hearing in deaf adult mice.^10^ Their findings support the existence of “ear glymphatic system” analogous to the brain or ocular glymphatic system. As known from the literature related to glymphatic system, the glymphatic clearance is delicate and can be adversely affected by many insults including age, sleep deprivation, hypertension, diabetes, and other factors. Extrapolating to the ear, it is possible that the inefficient clearance of waste products by an aging glymphatic system is contributory to the pathogenesis of presbycusis.

In conclusion, the elucidation of “ear glymphatic system” by emerging imaging technologies is an exciting possibility with important ramifications for many hearing‐related diseases. Further research is needed to map this system and understand its implications fully, which includes exploring its role in various auditory‐related conditions and its potential as a therapeutic target, as shown in Figure 1.

**FIGURE 1:**
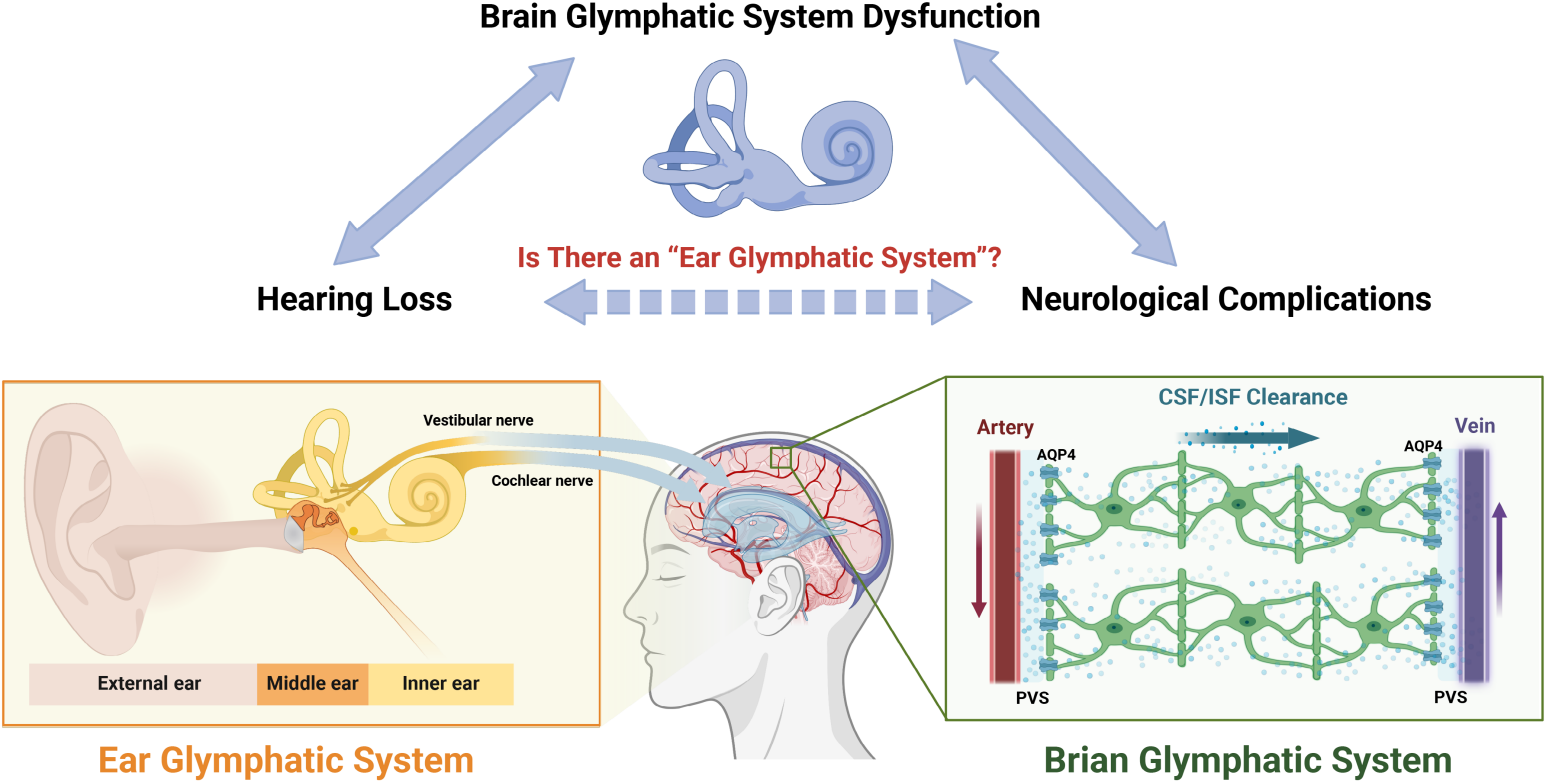
Ear may possess a similar glymphatic system, essential for clearing metabolic wastes, potentially linking auditory health with neurological functions. AQP4, Aquaporin‐4; PVS, Perivascular spaces.

## AUTHOR CONTRIBUTIONS

Yanlin Bi, Yunchao Yang, and Hong Wang drafted and revised the paper. Gaofeng Zhang and Rui Dong were responsible for drawing the picture. Rui Dong was responsible for the design and conception of the paper and revising the paper. All authors read and approved the final manuscript.

## CONFLICT OF INTEREST STATEMENT

The authors declare that there is no conflict of interest.

## Data Availability

Data sharing is not applicable to this article as no datasets were generated or analyzed during the current study.
